# PRE AND POSTOPERATIVE PH MONITORING AND WEIGHT LOSS ANALYSIS IN PATIENTS UNDERGOING GASTRIC PLICATION IN ASSOCIATION WITH FUNDOPLICATION

**DOI:** 10.1590/0102-6720201700040004

**Published:** 2017

**Authors:** Flávio Heuta IVANO, Luciana Pereira MESQUITA, Cristiane Megumi SIMAMURA, Gustavo Massaki KUWAKI, Julielli Taques COLMAN, Guilherme Mussi CAMPOS

**Affiliations:** 1 General Surgery and Obesity Service, Sugisawa Hospital, Curitiba, PR, Brazil

**Keywords:** Gastroesophageal reflux, Obesity, Fundoplication, Refluxo gastroesofágico, Obesidade, Fundoplicatura

## Abstract

*****Background*** ****:**

Obese patients with gastroesophageal reflux disease with pathological pH monitoring result may benefit from surgical treatment which is based on the fundoplication technique in association with laparoscopic gastric plication. The Nissen surgery is the gold standard for surgical treatment of gastroesophageal reflux disease, whereas laparoscopic gastric plication is a restrictive surgery that consists of the invagination of the greater curvature, resulting in weight loss.

***Aim*:**

To compare pre and postoperative pHmetry results and to evaluate weight loss in patients submitted to gastroplasty with fundoplication.

***Method*:**

Sixteen patients with class I body mass index with symptoms of gastroesophageal reflux with changes of stomach pH and/or erosive esophagitis seen in endoscopy were selected The evaluation of the weight and 24-h pH monitoring was performed preoperatively and postoperatively. The weight, body mass index, percentage of excess weight loss and DeMeester score of patients that underwent the surgery were evaluated pre and postoperatively.

***Results*:**

Regarding pH monitoring, the average preoperative DeMeester index was 28.7, which was followed by a significant postoperative average reduction to 2.8 (p<0,001). Regarding the weight reduction, the average of weight loss was 13.6 kg and body mass index of 5.3 kg/m^2^ (p<0.001). Furthermore, the average percentage of excess weight loss was 53.9% (standard deviation=26.2).

***Conclusion*:**

The combination of Nissen surgery and gastric plication is a viable procedure and appears to be an acceptable option for the treatment of gastroesophageal reflux disease in obese patients, especially patients with obesity class I.

## RESUMO

***Racional*:**

Pacientes que apresentam obesidade e doença do refluxo gastroesofágico comprovada com pHmetria, podem se beneficiar do tratamento cirúrgico que se baseia na união da técnica da fundoplicatura e gastroplicatura laparoscópicas. A operação de Nissen é o padrão-ouro para o tratamento cirúrgico; já a gastroplicatura laparoscópica é operação restritiva, cuja técnica é a invaginação intraluminar da parede da grande curvatura, o que resulta na perda de peso.

***Objetivo*:**

Comparar os resultados da pHmetria pré e pós-operatórias e avaliar a perda de peso em pacientes submetidos à gastroplicatura com fundoplicatura.

***Método*:**

Foram selecionados 16 pacientes com IMC em obesidade grau I, associado aos sintomas de doença do refluxo gastroesofágico, e com pHmetria alterada e/ou esofagite erosiva na endocopia digestiva alta. A avaliação do peso e da pHmetria de 24 h foi realizada no pré e pós-operatório. As variáveis foram: peso pré e pós-operatório, IMC, percentual da perda do excesso de peso e escore de DeMeester.

Dos 16 pacientes em relação à pHmetria a média pré-operatória do índice de DeMeester foi de 28,7, verificando-se na sequência redução significativa para média pós-operatória de 2,8 (p<0,001). Em relação ao peso, a média da redução foi de 13,6 kg e do índice de massa corporal de 5,3 kg/m^2^ (p<0,001). A média do percentual da perda do excesso de peso foi de 53,9%26,2±.

***Conclusão*:**

A associação da operação de Nissen com a gastroplicatura é procedimento viável e opção aceitável para o tratamento de doença do refluxo gastroesofágico em pacientes com obesidade grau I.

## INTRODUCTION

Obesity is a chronic disease with a complex and multifactorial etiology, which is characterized by an accumulation of lipids, resulting in metabolic changes. Obesity is also associated with an increased incidence of cardiovascular, neoplastic, gastrointestinal and respiratory diseases^1^ ^,^ ^4^. The World Health Organization (WHO) classifies obesity according to body mass index (BMI) in grade I obesity (BMI 30 to 34.9 kg/m²), grade II (35 to 39.9 kg/m²) and grade III (≥40 kg/m²). According to the Brazilian Institute of Geography and Statistics (BIGS 2008-2009), 49% of the Brazilian adult population is overweight, and 14.8% are obese^14^. Because of the chronic nature of obesity, its treatment is complex and multidisciplinary, which involves changes in lifestyle, and pharmacological and surgical treatment^1^.

The gastroesophageal reflux disease (GERD), which is often related to overweight and obesity comorbidity, is a chronic condition characterized by a retrograde flow of gastric contents (hydrochloric acid) and/or duodenal (bile salts and pancreatic enzymes) in the absence vomiting^5^ ^,^ ^15^. The most common typical symptoms include heartburn and regurgitation associated with postprandial worsening and decubitus positions. Other secondary atypical symptoms include epigastric pain, postprandial fullness, chest pain, nausea and dysphagia^7^ ^,^ ^15^. The pathophysiological mechanisms that support the emergence of this disease include hypotonia of the lower esophageal sphincter, hiatal hernia, increased abdominal pressure due to excess weight, previous abdominal plastic surgeries and inefficient motility of the esophagus^6^. The complementary tests for diagnosis of GERD consist of upper gastrointestinal endoscopy, contrasted radiological examinations, scintigraphy with technetium Tc 99m, esophageal manometry and 24-h pH monitoring^3^.

The relationship between obesity and GERD is a consensus by most researchers and its treatment is the goal of several studies worldwide and is demonstrated by the large number of comorbidities caused by the association of these illnesses^2^ ^,^ ^19^. According to Anand and Katz, the frequency of transient lower esophageal sphincter relaxation progresses with an increasing of BMI and abdominal circumference^2^. In obese patients, the internal pressure along the gastroesophageal junction increases reflux, and changes the sensitivity of the esophagus to acid^2^. Hiatal hernia, an increased intragastric pressure and gastroesophageal pressure gradient are also factors that predispose the gastroesophageal reflux^2^.

Regarding the treatment of GERD, the standard surgical treatment is the cruroplasty and total Nissen fundoplication which is a laparoscopic technique whose goal is to maintain the gastroesophageal junction in intra-abdominal position and restore the function of the cardia. For obesity, gastric plication is an under-development technique which may be a future alternative for the treatment of this condition^27^.

Laparoscopic gastric plication (LPG) is a restrictive method consisting in the reduction of intragastric space by invagination of the wall of the great curvature^27^. According to Mohammad Talebpour (2012), LPG has been improved for 12 years, and comparing with others restrictive surgeries the results in patients with morbid obesity show that it is an acceptable technique. Although weight loss is significant enough to almost match up to the results of other already established techniques, costs are lower in relation to bariatric surgery with staplers and complications were not more significant than in other procedures^27^.

Also in relation to LPG, the patient is independent in the postoperative period, with easy monitoring^27^. Moreover, a lower cost due to the unnecessary use of staplers or bands and short hospitalization period are very important factors for patients. A lower number of complications such as erosion and infection should be expected since this method is a more conservative procedure compared to other bariatric surgeries. However, if the patient does not maintain a proper lifestyle, the benefit of weight loss becomes questionable^27^.

It is known that obesity increases the incidence of GERD, the union of LPG and gastric plication can be an effective alternative for individuals with class I obesity and patients with GERD with pH changes. To prove the presence of acid in the esophagus lumen, 24-h pH monitoring is the best exam to confirm or rule out reflux in patients with clinical signs of GERD without esophagitis. In addition, it characterizes the features of reflux, recurrence of symptoms postoperatively and evaluates the effectiveness of medical or surgical treatment^3^.

The objective of the present study was to compare the pre and postoperative pH and to evaluate the weight loss in patients undergoing gastroplication with fundoplication.

## METHOD

This study was approved by the Research Ethics Committee at the Catholic University of Paraná, under protocol CAAE: 43711815.3.0000.0020.

In this cross-sectional study, patients undergoing LPG associated with laparoscopic fundoplication were evaluated between April 2012 and April 2016 at the Sugisawa Hospital, Curitiba, PR, Brazil.

All patients had symptoms of gastroesophageal reflux with pH changes and/or erosive esophagitis with increased BMI at upper gastrointestinal endoscopy. Patients with obesity class I without GERD, drug addiction or alcohol abuse, with mental illness that prevented the patient to understand and adhere to the type of treatment that should be followed postoperatively, severe clinical condition (e.g. unstable angina, recent heart attack, uncontrolled diabetes, cancer) that increases significantly the risk of surgery and age less than 18 or greater than 65 years, were excluded from this study.

In the preoperative period, the following tests were requested: ultrasound of the upper abdomen and blood tests - blood count, blood glucose, coagulation tests, creatinine, serum iron, lipid profile, evidence of liver and thyroid function, total protein and fractions, zinc, serum insulin, glycosylated hemoglobin, cortisol and parathyroid hormone. Esophageal 24-h pH monitoring was performed preoperatively and postoperatively. All patients enrolled in the present study underwent preoperative endoscopy and were in accordance with the research protocol and signed a free informed term of consent.

For surgical procedure, a pre-established technique of long Nissen fundoplication was used, and for the gastric plication, the technique described in 2010 by Ramos et al^12^ ^,^ ^18^ was used. The patients underwent general anesthesia, dorsal decubitus, with lower members opened and in proclive.

**FIGURE 1:**
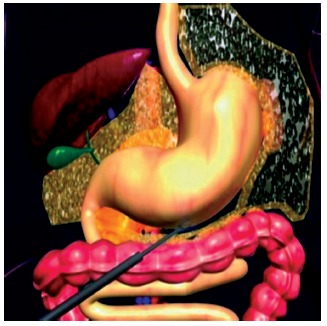
Greater gastric curvature Ligation

**FIGURE 2:**
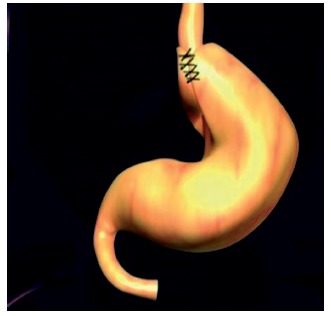
Fundoplication technical details

**FIGURE 3:**
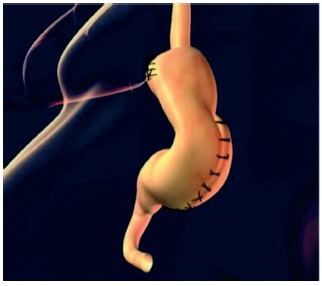
Gastroplication technical details

Laparoscopically, a ligation of vessels of large gastric curvature was made with ultrasound scalpel Sonosurg Olympus^®^ from 2 cm above the pylorus to the right crus of the diaphragm (Figure 1). Dissection of the abdominal esophagus was done, maintaining the extension of 5 cm, with correction of the hiatal hernia. Approximation of the diaphragmatic pillars using “X” suture with polyester 2-0 (Ehthibond^®^), calibrated with a nasogastric tube n^o^. 20 inside the esophagus and leaving a place of an opened pinch of dissection (2 cm). The fundoplication was performed with 360 degrees Nissen, passing the gastric fundus behind the abdominal esophagus and sutured with 2-0 polyester (Ethibond^®^) with four single sutures, the first two being fixed abdominal esophagus. Then, the forefront of longitudinal plication of the gastric body was made with separate points, with polyester thread 2-0 (Ethibond^®^), starting 2 to 4 cm above the pylorus and ending in the fundoplication valve, with a calibration with a probe Fouchet 32 inside the stomach (Figure 2). The suture was made near to the vessels of the small gastric curvature from the back wall to the front wall, invaginating the greater curvature. The second gastric plication plane with continuous sutures was made with an invagination of the first suture plan from the antireflux valve until the last separate point, near the pylorus. The gastroplication plan was made with single sutures, calibrated with 32 F Fouchet tube in the interior of the stomach, until it was observed a tubular aspect, similar to the final aspect of sleeve gastrectomy (Figure 3).

Patients underwent endoscopy after three months of surgery. They were prepared with an 8-h fasting before the exam. At the time of upper gastrointestinal endoscopy, they were positioned in the left lateral decubitus, and 100 drops of dimethicone were administered. Then, an oral sedation with performed with 1.5 to 2.5 mg/kg intravenously of 1% propofol. The procedure was performed with Olympus Model CV equipment 165. The videoendoscope was introduced through the mouth until reaching the esophagus. During endoscopy, the distensibility, the appearance of the mucosa, and presence of lesions and its regression (compared to the lesions observed preoperatively) were evaluated. The endoscopic appearance of longitudinal plication in the gastric body was also evaluated and as well as if the valve was continent, its length and its symmetry.

The 24-h pH monitoring was performed preoperatively and postoperatively for at least three months by means of a sensor located above the lower esophageal sphincter. The results were evaluated by measuring the DeMeester score, whereas the maximum limit of normal 95 percentile, whose value is 14.72^17^, was considered.

### Statistical analysis

Was conducted a statistical analysis of data through the computer program IBM SPSS v.20. Quantitative variables were described as mean, median, minimum and maximum values, and standard deviations. For qualitative variables, frequencies and percentages were presented. To evaluate the significant difference between before and after surgery a nonparametric test was used, the Wilcoxon´s test. The association between two quantitative variables was assessed by estimating the Spearman correlation coefficient.For overweight calculation, the current weight less ideal weight was used. The ideal weight was set from the Table of Metropolitan Life Foundation (MLF), which consist of different formulas for men (IW=61.2328 + [(H- 1.6002)x53.5433] and for women (IW=53.975 + [(H-1.524)x53.5433], where IW is the ideal weight and H the height in meters. The percentage of overweight is calculated by overweight x 100/ideal weight^10^ ^,^ ^11^ ^,^ ^23^. p<0.05 was considered statistically significant.

## RESULTS

In this cross-sectional study, 13 women and 3 men were included, totalizing 16 patients. The age of patients ranged from 18 years to 62 years.

### Description of DeMeester index

The mean of DeMeester preoperative index was 28.7 and the median was 22.5 with a maximum index of 90.4. As for the postoperative data were an average of 2.8, median 0.9 and a maximum value of 12.7. The mean reduction rate was 25.9, which resulted in statistically significant result (p<0.001, Table 1).

**TABLE 1:** DeMeester index, weight and BMI

Variables	n	Mean	Median	Minimum	Maximum	Standard-deviation	p
DeMeester pre	16	28.7	22.5	5.5	90.4	22.5	
DeMeester post	16	2.8	0.9	0.4	12.7	3.7	<0.001
DeMeester Reduction	16	25.9	21.3	5.0	77.7	20.4	
Preop weight (kg)	16	86.4	86.0	75.0	105.0	7.5	
Postop weight (kg)	16	72.8	72.3	56.0	91.0	10.4	<0.001
Weight reduction	16	13.6	14.0	4.0	22.0	5.5	
BMI preop	16	32.7	33.0	30.8	34.7	1.3	
BMI postop	16	27.4	27.6	23.0	31.6	2.7	<0.001
BMI reduction	16	5.3	5.1	1.8	9.7	2.4	

### Description weight, BMI and PEP

All patients undergoing surgery had a significant weight loss postoperatively, which was evaluated on a return visit. The mean of preoperative weight was 86.4 kg and a minimum of 75 kg to a maximum of 105 kg. There was a statistically significant reduction of weight, with a mean of weight loss of 13.6 kg (p<0.001).

The median of preoperative BMI was 32.7 kg/m^2^ and after surgery was 27.4 kg/m^2^. Median BMI reduction was also statistically significant, and the reduction of BMI was 5.3 kg/m^2^ (p<0.001, Table 1).

The mean of %EWL was 53.9% (median 49.8%), ranging from a minimum of 16.9% to 97.4% (Table 2).

**TABLE 2:** Change in excess weight loss (%PEP)

Variable	n	Mean	Median	Minimum	Maximum	Standard-deviation
%EWL	16	53.9	49.8	16.9	97.4	26.2

## DISCUSSION

There are many available bariatric procedures that are used for weight reduction^17^. However, there is no consensus about performing surgery in obese patients in class I^9^. Considering that obese patients have a strong relationship with GERD, studies have been developed to demonstrate the combined treatment for both of these morbidities, using the combination of gastric plication and fundoplication techniques. These techniques, however, had already been separately described to reduce the symptoms of GERD and weight loss, respectively.

In a prospective longitudinal study with 12 years of experience in laparoscopic gastric plication technique, Talebpour et al. (2012) demonstrated advantages such as easy postoperative follow-up, lower cost by not using staplers, low complication rate (0.6%), low rate of reoperation (1%) possibility of reversal and during the follow-up, only 31% of patients had regained weight. Given these benefits, a strong interest from the medical community to use this surgery have arisen, which proved to be efficient and conservative for weight reduction^27^.

In Brazil, the technique was first described by Fusco et al (2007), in an experimental study with Wistar rats, which had greater weight loss using the greater curvature invagination technique (the same used in LGP) against the wall of invagination anterior gastric^13^.

Considering the surgical treatment options for GERD, the total fundoplication or 360 degrees Nissen surgery is considered the gold standard^22^. This technique proved to be superior when compared to partial fundoplication. The advantages of Nissen surgery are lower dysphagia^25^, hiatal hernia and esophagitis index, and the increase of lower esophageal sphincter pressure. It is also been reported weight loss of approximately 4 kg in three months, and no trend to regain weight in the 1^st^ year after surgery, which was probably due to early satiety described by some patients^8^ ^,^ ^20^.

Considering the applicability of the two techniques and analysing the group weight loss and DeMeester reduction in this study, the association of fundoplication with LGP not only treats GERD, but also provides the patient with class I obesity, which has no formal indication for surgical treatment, the benefit of losing weight excess, improving their quality of life. Thus, the sample of the present study showed a reduction of DeMeester index values and statistically significant weight loss (p<0.001). It is noteworthy that the lifestyle change, with nutritional education and practice of regular physical exercise was guided as a complementary treatment, influencing satisfactory result and consequently the success of the established goals. Lee et. al. (2014) conducted this association of procedures in morbidly obese patients with GERD. The result was satisfactory with regard to loss of excess weight (EWL%) with a mean of 24.6±9 kg (46.7%)^18^. Khazzaka and Sarkis (2013) analyzed the same profile of patients, achieving an average of 10±4 kg (58%), but using the plication of the gastric body. In the present study, which examined patients with obesity class I with GERD, there was an average of 53.9% EWL (median 49.8%), confirming the success of the technique since the bariatric procedures are considered effective if the patient reaches at least 50% loss of excess weight^21^.

There are some limitations in the present study that should be considered. It appears that the postoperative follow-up period for evaluation of pH monitoring, weight loss and upper gastrointestinal endoscopy, was variable and low. Regarding 24-h pH monitoring, which is an uncomfortable examination, it was found difficulty convincing the patient, virtually asymptomatic postoperatively, to perform the examination, influencing the sample size. The loss of weight, which was assessed very early, might not have reached the maximum loss of weight possible. Still, one patient did not do the upper gastrointestinal endoscopy postoperative, making it impossible to analyze their endoscopic changes. Another limitation was the analysis of weight loss in a quantitative manner, without taking into account the influences of changing lifestyle. And because the LGP is a recent technique, they were not found enough studies in the literature for better comparison.

Finally, like any surgical procedure, this operation may have complications such as micro-perforations, obstructions, adhesions that cause nausea, vomiting, drooling and intrabdominal bleeding^8^ ^,^ ^23^ ^,^ ^27^. Furthermore, as a new technique with few studies with few samples, the patient may be vulnerable to risks not yet described. Therefore, more studies are needed with larger numbers of patients and longer follow-up to improve the level of evidence.

## CONCLUSION

The association of gastric plication and fundoplication demonstrated both weight loss and reduction of DeMeester index.
